# Genome-wide sequence and functional analysis of early replicating DNA in normal human fibroblasts

**DOI:** 10.1186/1471-2164-7-301

**Published:** 2006-11-29

**Authors:** Stephanie M Cohen, Terrence S Furey, Norman A Doggett, David G Kaufman

**Affiliations:** 1 Department of Pathology and Laboratory Medicine, University of North Carolina, Chapel Hill, North Carolina 27599, USA; 2 Institute for Genome Sciences and Policy, Duke University, Durham, NC, 27708, USA; 3 Bioscience Division, Los Alamos National Laboratory, Los Alamos, New Mexico 87545, USA

## Abstract

**Background:**

The replication of mammalian genomic DNA during the S phase is a highly coordinated process that occurs in a programmed manner. Recent studies have begun to elucidate the pattern of replication timing on a genomic scale. Using a combination of experimental and computational techniques, we identified a genome-wide set of the earliest replicating sequences. This was accomplished by first creating a cosmid library containing DNA enriched in sequences that replicate early in the S phase of normal human fibroblasts. Clone ends were then sequenced and aligned to the human genome.

**Results:**

By clustering adjacent or overlapping early replicating clones, we identified 1759 "islands" averaging 100 kb in length, allowing us to perform the most detailed analysis to date of DNA characteristics and genes contained within early replicating DNA. Islands are enriched in open chromatin, transcription related elements, and Alu repetitive elements, with an underrepresentation of LINE elements. In addition, we found a paucity of LTR retroposons, DNA transposon sequences, and an enrichment in all classes of tandem repeats, except for dinucleotides.

**Conclusion:**

An analysis of genes associated with islands revealed that nearly half of all genes in the *WNT *family, and a number of genes in the base excision repair pathway, including four of ten DNA glycosylases, were associated with island sequences. Also, we found an overrepresentation of members of apoptosis-associated genes in very early replicating sequences from both fibroblast and lymphoblastoid cells. These data suggest that there is a temporal pattern of replication for some functionally related genes.

## Background

A highly organized and strictly controlled process is necessary to accurately replicate the six billion base pairs of DNA that are tightly packaged within the confined volume of the human diploid nucleus. In order to accomplish this task, DNA replication is initiated at distinct sites in the genome as cells enter the S phase [1]. The regulation of initiation is important because once the S phase begins, a cascade of events results in the successive activation of new replication clusters in a temporally and spatially ordered manner [2,3]. The order of activation of the estimated 30,000 replicons in the human genome [4] is maintained through successive cell cycles [5] and is tissue specific [5,6]. It is not yet fully understood how this sequential firing of replicons, resulting in the orderly progression through S phase, is regulated in human cells, but disruption of this process can have far reaching consequences that include acquisition of genetic instability leading to cancer.

The distribution of early and late replicating DNA is seen cytogenetically when metaphase chromosomes are Giemsa banded. It has been known for some time that Giemsa-negative or reverse (R) bands replicate early in S phase while Giemsa-positive (G) bands replicate late [7-9]. While the exact molecular basis for chromosomal banding is not understood, it has been proposed to be related to differences in chromatin condensation [10], the arrangement of scaffold-loop structures [11] or differences in GC content between neighboring regions [12]. Indeed, R bands were reported to have a higher GC content [9] and have a higher density of genes [13,14] and CpG islands [15] than G bands. Short and long interspersed nuclear elements (SINE and LINE, respectively) were also found to be unevenly distributed; a higher SINE frequency was found in R bands while LINE elements were disproportionately found in G bands [16,17].

The distribution of the above-mentioned sequence features of early replicating DNA in the giemsa-negative bands (R-bands) were determined using cytogenetic methods. More recently, studies by Woodfine et al. [18,19] confirmed the cytogenetic data, utilizing a microarray approach. Microarrays containing human genomic sequences were used for comparative hybridization of DNA isolated from S phase and G_1 _cells. Using this methodology, they found a positive correlation between early replication and CpG islands, GC content, expressed genes, and Alu repeats (a member of the SINE class of repeats). A negative correlation was found with LINE elements [19]. White et al. [20] who found enrichment of transcriptionally active (but non-protein encoding) regions in early replicating sequences from chromosome 22 used a similar approach. Jeon et al. [21] investigated replication timing on chromosome 21 and 22 using high density genome-tiling arrays. Gene density, exon density, and gene expression were all highly correlated with early replication in their study. They hypothesized that an open chromatin environment, as reflected by high exon density separates early replicating DNA from that replicating later. In another study, Gilbert et al. [22] separated compact and open chromatin fibers and studied their genomic distribution using microarrays containing a sub-set of genomic sequences. They found a positive correlation between open chromatin and early replication according to results from low-density genome-wide arrays. This correlation disappears however, when comparing chromatin structure data and time of replication from high-density arrays for chromosome 22.

We have taken a different approach to study characteristics of DNA that replicates early in the S phase. We reported previously the construction of a cosmid library enriched in early replicating sequences from normal human fibroblasts [23]. In the present study, we end-sequenced and mapped clones from this library, identifying "islands" where clones were adjacent or overlapping. We then verified that our island sequences exhibited the features of early replicating DNA reported by others (i.e., high GC, Alu, and gene content, and open chromatin structure) as well as active promoters. Also, we determined more precisely the time of replication for selected islands and compared them to data reported by Woodfine et al. [18,19]. We performed a more extensive analysis of repetitive sequences in early replicating DNA, including long terminal repeats (LTRs), DNA transposons, and inverted, tandem, and simple repeats. We also identified genes associated with islands of early replicating sequences and found an enrichment in *WNT *genes and DNA glycosylases and, using the GOstat program, an overrepresentation of genes involved in apoptosis. Finally, we analyzed genes that overlapped markers tested by Woodfine et al. [18,19] for replication timing and found that apoptotic genes also replicate very early in lymphoblastoid cells.

## Results and discussion

### Synchronization of normal human fibroblasts and library construction

The construction of a subgenomic cosmid library enriched in early replicating sequences has been previously described [23]. Briefly, normal human fibroblasts (NHF1) obtained in this laboratory from neonatal foreskin [24] were synchronized to early in the S phase by a combination of confluence arrest, followed by replating at lower cell density in the presence of aphidicolin. The DNA polymerase inhibitor aphidicolin allows for the initiation of DNA replication, but dramatically slows replication fork progression [25,26] without altering the order of gene replication [27]. Bromodeoxyuridine (BrdUrd) was added to aphidicolin-containing medium to label DNA replicated as cells entered the S phase. Nuclear DNA purified from cells harvested 24 h after replating was partially digested with Sau 3AI, and hybrid-density DNA was separated in CsCl gradients. The purified early-replicating DNA was cloned into the sCos1 cosmid vector. Clones were transferred individually into the wells of 96 microtiter plates (9,216 potential clones).

**Figure 1:**
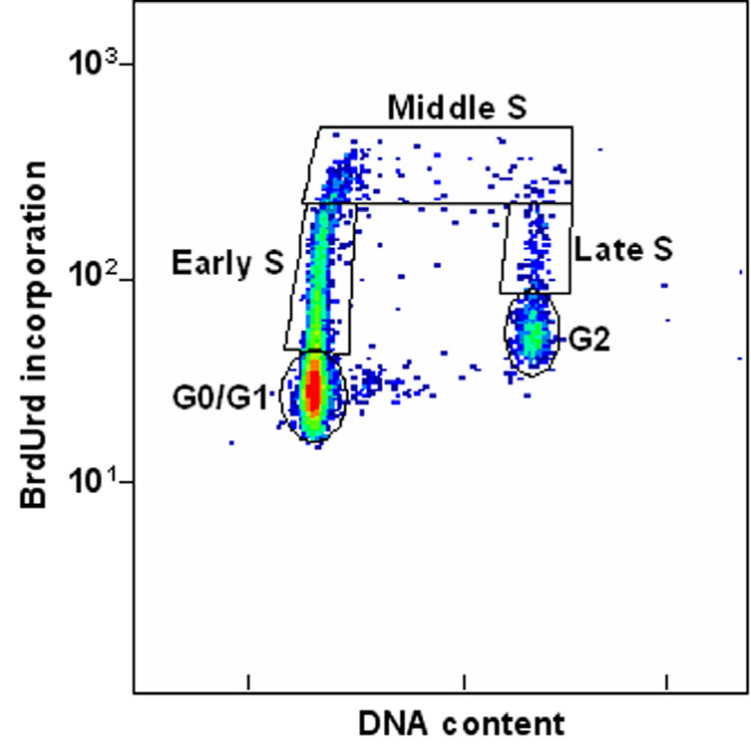
**Flow cytometry of synchronized NHF1 cells**. Cells were grown to confluence arrest and replated at low density in the presence of aphidicolin and BrdUrd as described. After 24 hrs cells were collected and analyzed by flow cytometry. The ordinate of this graph shows BrdUrd incorporation as indicated by fluorescently-labeled anti-BrdUrd antibody and the abscissa shows DNA content as indicated by propidium iodide staining. The boxes outline areas that were analyzed for cell number.

Figure 1 shows a typical flow cytometry analysis of NHF1 cells after 24 hrs in aphidicolin and BrdUrd. This profile shows that with this method of synchronization, most cells are in G0/G1 and at the G1/S border. If we look exclusively at the cells containing BrdUrd in the experiment shown in Figure 1, 80.4% were in early S, 13.3% were in middle S, and 6.3% were in late S. Since only BrdUrd-labeled DNA was used to generate the cosmid library, these flow cytometry values give us an approximate S phase distribution of DNA in our islands. This figure illustrates that most of the DNA used to create the cosmid library was isolated from cells that were in early S phase. It also shows that although the cells were well synchronized a small but measurable fraction of cells were in middle and late S. The presence of these middle and late S phase cells in the synchronized population are probably the result of the approximately 3% of cells that are still cycling in the confluence arrested cultures. We have found that this number can be reduced by increasing the amount of times held at confluence arrest. Increased time at confluence arrest however, will result in less cells cycling after they are replated at lower density (unpublished observations).

### Identification of islands

**Figure 2:**
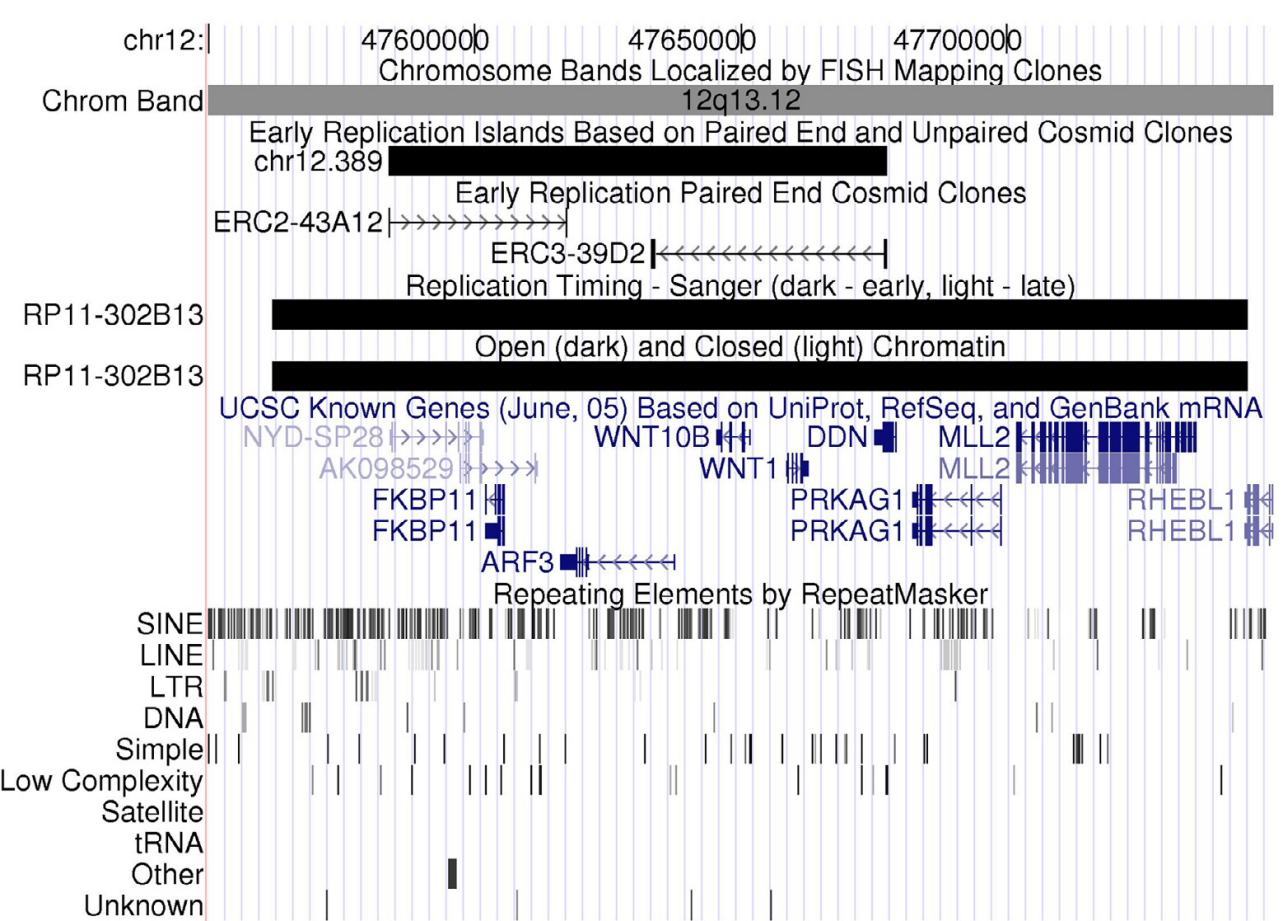
**Island represented in mirror of UCSC Genome Browser**. The early replication island *chr12.389 *is displayed as an annotation track in a mirror copy of the UCSC Genome Browser and is available at [29]. The cosmid clones that support this island, ERC2-43A12 and ERC3-39D2, are also shown below. This island overlaps a BAC clone, RP11-302B13, for which both replication timing [18, 19] and an open chromatin status [22] are available. The dark boxes indicate that this region was found to replicate early (timing ratio = 2.00) and to have an open chromatin conformation (log2 open = 2.73). The 200-kb region displayed is gene dense, including two *WNT *genes, is highly enriched in SINE repeat elements, and shows a paucity of LINE, LTR, and DNA transposons.

End sequencing of 9216 cosmid clones from a library enriched in early replicating DNA yielded high quality sequences for both ends of 6370 clones; reliable sequence for a single end was obtained for another 1521 clones, for a total of 7891 clones with at least one end sequenced. The remaining 1140 clones produced only poor quality traces or showed evidence of contamination. Successful reads were aligned to the genome as detailed in the Methods section, resulting in the placement of 7614 (96.5%) clones, 5818 using both ends. Due to segmental duplications and repetitive sequences at their ends, some clones could not be placed uniquely and were discarded, leaving 7455 (94.5%) clones for further analysis. We theorized that clusters of clones or "islands" are the result of redundant sampling of early replicating regions and provide an effective mechanism for screening out most non-early replicating clones that may be the result of a small amount of total genomic DNA contaminating the BrdUrd-labeled DNA recovered from density gradients. We looked for evidence of clustering of early replicating cosmid clones by defining early replicating islands as regions in the genome ≤ 175 kb in length, where two or more clones were localized, with a maximum distance of 100 kb between clones. We found 1759 islands that met these criteria; they had an average island size of ~100 kb, and included 4250 clones. We noticed that the average size of these islands is in close agreement with the average size of a replicon [4], and it is likely that sequences within each island replicate at the same time. An example of one island, as displayed in a mirror copy of the UCSC browser [28] available at Duke University [29], is shown in Figure 2. Islands were found on every chromosome, but were not equally distributed. Table 1 shows the relative enrichment or under-representation of island DNA based on the genomic percentage of each chromosome. Chromosome 19, which comprises 2.25% of the genome, was the most enriched, with 4.4% of the island DNA originating from this chromosome. In contrast, sequences from the X and Y chromosomes had the lowest representation in island DNA. This distribution of islands corresponds well with the mean replication timing ratios determined for each chromosome reported by Woodfine et al. [19] for lymphoblastoid cells. As shown in Table 1, the five chromosomes with the highest percentage of island DNA corresponds to five of the six chromosomes with the earliest replicating timing as determined by Woodfine et al. [19].

It has been previously shown in mammalian cells that chromosomal bands that do not stain with Giemsa tend to replicate early while bands that do stain replicate late [7-9]. Using the estimated chromosomal band positions available in the UCSC Genome Browser [30], the percentage of early replicating island DNA in each band class was determined. There is a significant enrichment (p < 0.001, χ^2 ^test) in G-negative bands with ~59% of island DNA in this band class as compared to 45% of total sequenced genomic DNA in these bands. In contrast, only 17% of islands were found in the two darkest staining and heterochromatic late-replicating bands, while nearly 33% of genomic DNA is contained within these bands. When we examined those markers used by Woodfine et al. [18,19] that comprise the earliest replicating 25% of markers tested (markers with replication timing ratios > 1.75), we found that ~70% were located in G-negative bands. From these studies with higher resolving power than previous cytogenetic analyses, we concluded that while early replicating sequences are found predominantly in G-negative bands, a sizeable portion is not.

### Further validation of replication timing using synchronized normal human fibroblasts

Replication timing data in the Woodfine et al. studies [18,19] mentioned above was determined in lymphoblastoid cells while our library was derived from normal human fibroblasts. In the Woodfine et al. studies, genomic DNA from S phase lymphoblastoid cells was hybridized to microarrays containing clones that were distributed at ~1 Mb intervals across the genome, at a resolution of ~70 kb on chromosome 22 [19], and ~94 kb on chromosome 6 [18]. The hybridization signal from S phase cells was compared to the hybridization signal for DNA isolated from G_1 _cells to determine the replication timing ratio. This ratio reflects the copy number of each clone in the S phase DNA, and ranged from ~1.0 to 2.0, with 2.0 representing the earliest replicating sequences tested. We decided to test several islands using a quantitative PCR assay [31,32] to check for concordance between their replication time in fibroblasts and the replication timing ratio determined by the Woodfine group in lymphoblastoid cells [18,19]. A total of 20 markers from 10 islands were tested (Table 2). To provide a robust comparison, islands containing three or more clones were chosen to cover a variety of replication timing ratios as determined by Woodfine et al. [18,19] with the high-resolution data for chromosomes 6 and 22. For each primer set (Table 2), an equal amount of replicating DNA from samples representing seven 1-h windows of the S phase was tested and the approximate time of replication was determined as described in the Methods section.

**Figure 3:**
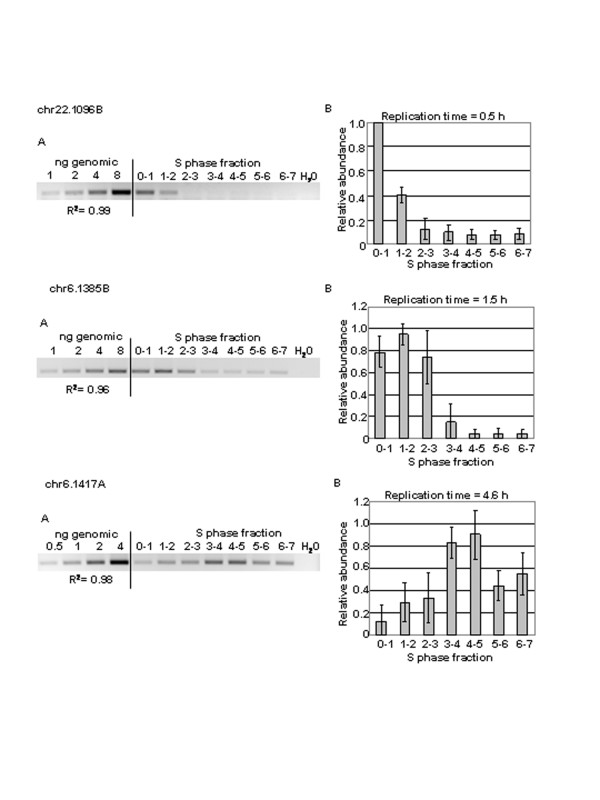
**Determination of the timing of replication of genetic markers by PCR**. The three composite panels illustrate the procedure used for determining which of seven samples of DNA, each replicated at a different 1-h period of the S phase, was enriched for copies of a particular marker. DNA replicated at the indicated 1-h intervals of the S phase of synchronized fibroblasts was labeled with BrdUrd and isolated by CsCl centrifugation. In each panel, A: Inverted contrast image of PCR products stained with ethidium bromide after gel electrophoresis. The same primer set was used to amplify increasing amounts of genomic DNA and DNA replicated during each of the first 7 h of the S phase; a control with no template DNA was included in every PCR experiment. The R^2 ^value for each standard curve that was obtained by plotting the signal intensity of the bands of PCR products in the gel above versus the amount of genomic DNA, is shown. B: Bar graph illustrating the abundance of the marker in each of the seven 1-h samples of the S phase. Results show the average of two synchronizations that were each tested at least twice. Vertical bars indicate the standard deviation for each time point. Relative abundance was calculated from the linear regression equation of the standard curve and expressed as a percentage of the S phase fraction with the highest value. Replication times were calculated as described in the Methods section. The three panels shown illustrate the results obtained with markers that replicate in the first hour of the S phase (primer chr22.1096B), during the second hour (chr6.1385B), and at 4.6 hrs into S phase (chr6.1417A).

Figure 3 illustrates the primary data for three markers; the results for all markers tested are listed in Table 2 and are the average of PCR analyses from two separate synchronizations. We were able to assign replication times for 17 of the 20 markers, with good agreement between the two synchronizations. Only one marker, chr22.1102A, gave results that did not allow for replication time assignment. In the samples from both synchronization experiments, this chromosome 22 marker was enriched in DNA replicated during the 1^st ^and the 5^th ^hr of the synchronous S phase. At another island, (primer sets chr6.1401A and B) there was approximately 1 hr difference in replication time between the two synchronizations that were tested indicating that there were some experimental differences in the two isolations of replicating DNA. Our ability to determine a specific replication time for nearly all of the markers that we tested is consistent with our previous studies on chromosome 1p36 [32], and in studies reported by others [33-35], but is in sharp contrast to the results of replication timing experiments reported by Jeon et al. [21]. In replication timing studies reported by this group they found that 60% of the sequences that they tested replicated throughout the S phase. Looking at their data, we did observe that there seems to be something unusual about the replication dynamics in the HeLa cells that they used. We noticed that the S phase in these cells lasted 10 hrs, with a peak of DNA synthesis found at six hrs. In our experience with normal diploid human fibroblasts, S phase lasts about 8 hrs, peaking at about 4 hrs into S phase [32]. Indeed, the possibility that the pan-replication pattern that was found in 60% of the sequences that were tested Jeon et al. was a characteristic of cancer cells was pointed out by the authors themselves. It should be noted however, that not all cancer cells have this pan-replication pattern since Tenzen et al. [34] were able to obtain discrete replication times for markers in myeloid leukemia cells.

We tested nine markers from five islands that overlap with sequences that have Woodfine replication timing ratios of 1.72 and higher [18,19]. Seven out of nine of these markers replicated in the first hour of S phase, the other two replicated in the second hour indicating that there was good agreement in replication time for fibroblasts and lymphoblastoid cells in the earliest replicating sequences. White et al. [20], who compared their fibroblast replication timing on chromosome 22 to the Woodfine et al. data [19], found similar results. Also, Janoueix-Lerosey et al. [36], who studied replication timing in neuroblastoma cells found that 60% of early replicating sequences also replicated early in lymphoblastoid cells.

Although our library is enriched in early replicating DNA as mentioned previously, it does contain a small fraction of middle and late replicating sequences. For example, we used two PCR primer sets to test two points in an island that overlaps a Woodfine marker with a replication ratio of 1.51. In our assay, we found that this island replicates between 4.6 and 4.8 hrs, just past the middle of S phase. Another island, also tested for replication timing, was one of the few that were very late replicating, with a replication time of 5.6 and 5.5 hrs and overlapping markers with Woodfine replication ratio of 1.36 and 1.25. While these markers correlated well with data from lymphoblastoid cells, this did not hold true for all markers tested in fibroblasts. For example, clone island chr22.1110 overlaps markers with Woodfine replication ratios of 1.55 and 1.55, but testing of two PCR markers in this island indicated that in fibroblasts it replicated in the first hour, not in the middle of S phase. Additionally, PCR marker chr6.1395C, which is located in a region with a replication timing ratio of 1.58, replicates at 5.4 hrs, as do primer sets A and B from that same island that have Woodfine replication ratios of 1.36 and 1.25. These differences in timing might be the result of differences in cell type and could reflect differences in gene expression and/or chromatin condensation [22] between lymphoblastoid and fibroblast cells.

### Distribution of repetitive elements in early replicating DNA

As mentioned previously, SINE repetitive elements were found to be enriched in early replicating regions while there was a paucity of LINE elements in these regions, as compared to the genome as a whole. Islands were evaluated for the presence of these and several other repetitive elements as compared to all non-overlapping 100 kb windows across the genome. Table 3 lists those elements whose content is significantly different in islands compared to the genome as a whole (as described in the Methods section). Both of the major SINE elements, Alu and mammalian-wide interspersed repeats (MIR), and L1 LINE elements were found to be distributed differently between clone islands and genomic DNA, while L2 elements were not. Four major classes of repeats that had not been investigated previously for replication timing distribution were examined here. LTR retrotransposons (mammalian LTR [MaLR], endogenous retrovirus 1 and L [ERV1 and ERVL]), DNA transposons, and low complexity repeats, were found less frequently in islands, similar to L1 elements. Alternatively, tandem repeats were found more frequently in islands. We then specifically looked at islands that overlapped markers distributed at ~1 Mb across the genome that were tested by Woodfine et al. [19] for replication timing, especially those that replicated early in the S phase. As shown in Table 3, the trends in repeat feature distributions found for all islands are even more pronounced in the islands that overlap with markers having replication timing ratios of 1.65 and higher. For example, Alu sequences are found most frequently in islands overlapping markers with replication timing ratios ≥ 1.85.

As mentioned above, tandem repeats, which included micro- and mini-satellites, were found enriched in islands. It has been established previously that tandem repeats are distributed in a nonrandom manner, with most being found in non-coding DNA and in both intergenic regions and introns [37]. This is probably due, with the exception of tri- and hexa-nucleotide repeats, to selection against frameshift mutations [37]. An excess of tandem repeats in early replicating DNA however, has not been reported previously. Some of this enrichment may be linked to the other repetitive elements that are enriched in early replicating sequences. For example, Alu repeats often contain microsatellite-like regions at their 3' ends [38]. Since we found an enrichment of Alu sequences in islands, this could explain some of the enrichment of certain types of A-rich microsatellite structures. To determine whether A-rich microsatellites were responsible for the enrichment, we looked at the distribution of individual classes of tandem repeats in islands. This included mono-, di-, tri-, tetra-, penta-, and hexa-nucleotide repeats, as well those with a periodicity of greater than six bases [see Additional file 1]. We found that all classes of tandem repeats, except di-nucleotides, were present at a higher level in islands than in the genome as a whole. Di-nucleotide repeats were actually found to be slightly depleted in islands. It is not clear why di-nucleotide repeats do not follow the same early replicating trend as other tandem repeats. Changes in the length of and presumably the establishment of microsatellites are thought to be caused primarily by DNA slippage during replication [39]. This mechanism would involve increases in the length of short, and decreases in the length of long tandem repeats [40]. Most of these changes are corrected by the mismatch repair pathway [41]. It is possible that there are differences in the process that causes slippage mutations [40], or the ability to repair, the different classes of tandem repeats. These differences would presumably be influenced by the genomic features associated with early replicating sequences.

### Open chromatin in early replicating regions

Using the same 1 Mb clone microarray as Woodfine et al. [19], Gilbert et al. [22] determined ratios for the enrichment of open (positive) regions of chromatin for each marker. Sites that were depleted in open chromatin sequences were considered to be regions of more compact (negative) chromatin. Lymphoblastoid cells were used in the Gilbert et al. [22] study and chromatin conformation most certainly differs between fibroblasts and lymphoblastoid cells in select regions. Nonetheless, we hypothesized that the overall chromatin conformation should be similar in both cell types. Gilbert et al. [22] previously reported a good correlation between open chromatin and early replication, based on the replication timing results of the 1 Mb array reported by Woodfine et al. [19]. They note, however, that except in the regions with the highest enrichment in open chromatin, this correlation diminishes when chromatin data is compared to the high-resolution replication data on 22q. Gilbert et al. [22] suggested that, since there were a multitude of sites that were not enriched in open chromatin but were nevertheless early replicating, the two physiological characteristics (open chromatin and early replication) were not functionally related.

We investigated islands that overlapped the same 1 Mb markers tested by Gilbert et al. [22] for chromatin confirmation and determined that over two-thirds of islands were found in an open state (log2 open > 0, as determined by Gilbert et al. [22]) as detailed in Table 4. In addition, among islands that overlapped markers with replication timing ratios ≥ 1.85 (as determined by Woodfine et al. [19]), over 90% corresponded to regions enriched in open chromatin. In contrast, less than half of all genome-wide 100 kb windows that overlapped array markers were determined to be in an open state. To assess how well an open chromatin state predicts early replication, we analyzed the 100 markers that were determined by Gilbert et al. [22] to have the highest log2 open ratios and found that 38 directly overlapped an island. Another 23 overlap a single early replicating clone, and 15 were within 50 kb of an island or clone. In contrast, among the 100 arrayed markers with the lowest log2 open ratios, only eight overlapped an island, 19 overlapped a single early replicating clone, and nine were within 50 kb of an island or clone. Over half of this mostly closed chromatin group was greater than 100 kb from the nearest early replicating clone. These data would seem to indicate, therefore, that the most open sequences are correlated with early replication, in agreement with the results reported by Gilbert et al. [22] for the 1 Mb array markers.

We also investigated regions of 22q where a total of 28 islands overlapped markers from the high-resolution array where the Woodfine et al. [19] data suggested an early replication time (replication ratio ≥ 1.75) and where chromatin data was also available [22]. Since our islands average 100 kb in length and the spacing of clones on this array averages 70 kb, it is not unusual for an island to overlap more than one array marker. Together, the 28 islands overlapped 20 markers found to be in a more closed chromatin state (log2 open < 0) and 26 markers in an open state (log2 open > 0). Based on this data using markers from the high-resolution array, there is clearly little correlation between early replication and open chromatin as noted by Gilbert et al. [22]. However, this lack of correlation could be attributed to a difference in the cell types, and thus chromatin states, at these locations in lymphoblastoid and fibroblast cells. Our data from chromosome 22 indicates another possible explanation. We found that 20 of the 28 islands (71%) overlapped at least one marker with log2 open > 0, but some also overlapped an additional marker with log2 open < 0. An example of this type of distribution occurred with island chr22.1090, which was defined by 6 separate early replicating clones, is 140 kb in length, and overlapped three arrayed markers with log2 open chromatin ratios of -0.538, 0.435, and 0.951. It is possible in these instances that an origin of replication that fires early in the S phase may be contained in the region of open chromatin, and fork progression from this origin extends into the nearby regions of more compact chromatin. Identification of replication origins and replicon boundaries in these regions will help to determine if this is indeed the case.

### Transcription and early replication

Islands were evaluated for features related to transcription. Islands had a higher gene density, and were enriched in transcribed and translated sequences as compared to the whole genome (Table 3). A correlation between early replication and actively transcribed regions has been previously established [19,42]. It is, therefore, not surprising that CpG islands, CpG content, and GC content are also found at higher densities in early replicating DNA, as each of these parameters are related to gene density. Likewise, we found that islands had greater amounts of evolutionarily conserved elements ([43]; [see Additional file 2]) that are also predominately associated with gene sequences.

In a recent report, Kim et al. [44] evaluated the transcriptional status of promoters in human fibroblasts (IMR90 cells) by analyzing these sequences for the binding of the RNA polymerase II preinitiation complex. Here, we evaluated the distribution of active promoters [44] in early replicating DNA islands. As expected, active promoter density is greater in the islands as compared to the genome as a whole. Again, differences in feature content are even greater when considering only the subset of islands that overlap markers tested by Woodfine et al. [19] with replication timing ratios of ≥ 1.65 (Table 3).

### Functional analysis of early replicating genes

Although it has been known that expressed genes, especially house-keeping genes, replicate predominately in the first half of S phase [42], these studies have not defined with great accuracy how early in S phase they replicate nor which genes are replicated in successive cohorts. In the present study, we were able to examine this question with greater precision. We looked at genes associated with islands and found that nine (47%) of the 19 *WNT *genes (wingless-type MMTV integration site family) were present. The nine island-associated WNT genes were located in eight islands found on eight different chromosomes. This family of genes is involved in stem cell regulation, wound healing in dermal fibroblasts, and is associated with cancer in many tissue types [45]. Islands were enriched in genes associated with DNA repair, especially those related to the base excision repair pathway [46,47]. These include four of ten known human DNA glycosylases (*MBD4, NTH1, MPG *and *NEIL2*), and several genes involved in the repair of apurinic/apyrmidinic sites (*XRCC1, POLB, POLL, PARP1, FEN1*, and *RPA3*). *MGMT*, whose gene product directly repairs O^6^-methylguanines, is also associated with a library island. A complete list of genes associated with library islands can be found in Additional file 3.

To evaluate further the potential function of genes that were associated with the islands we used the GOstat program [48,49]. This program uses the information in the Gene-Ontology (GO) database to generate statistical information on which categories of genes are over or underrepresented in a given list of genes. Results are shown in Table 5. From a total of 3265 unique island-associated genes UCSC Known Genes dataset [29], 1975 genes were assigned to functional categories. Several categories were over or underrepresented in comparison to the full UCSC Known Genes dataset. Overrepresented categories included cell death/apoptosis and the frizzled-2 (WNT) signaling pathway. Pro-apoptotic genes found associated with the library function in all aspects of the apoptotic process, and include *FAS, TRAF7, BID, BAD, BAX, CASP3*, and *CASP8*. Anti-apoptotic genes included *BCL2, TRAF1*, and *TRAF2*. The overrepresentation of genes in both the WNT and apoptotic pathways in islands is interesting since these two pathways function together for morphogenesis during development and for tissue homeostasis in differentiated cells (reviewed in [50]). For example, some WNTs can inhibit apoptosis through the β-catenin/T-cell factor transcription mediated pathway. This inhibition of apoptosis has been shown for a variety of cell types including fibroblasts [51]. Underrepresented gene categories included rhodopsin-like and olfactory receptors. These are tissue specific genes that are not known to be expressed in dermal fibroblasts and therefore, were not expected to be found in early replicating DNA from these cells.

Our previous studies showed that cells in the earliest part of the S phase of the cell cycle are most vulnerable to malignant transformation by chemical carcinogens [52-54]. We hypothesized that the vulnerability of cells at the start of the S phase to carcinogen-induced changes that lead to cancer is due to the increased probability of mutating a relevant genetic target (whether a cancer gene or a regulatory region) during its replication at the time of carcinogen treatment early in the S phase. The finding that apoptotic, WNT genes, and DNA repair genes replicate in this compartment of the S phase, therefore, provides novel information on possible targets for carcinogenesis that are consistent with previous reports for common alterations in cancer.

We also examined the distribution of gene ontology categories for replication timing data reported for lymphoblastoid cells by Woodfine et al. [18,19]. We found that markers used in these studies [18,19] overlapped with ~9000 genes in the Known Gene dataset. For GOstat analysis, markers tested by Woodfine et al. [18,19] were separated into three bins based on replication timing ratios: > 1.75 (very early; 3213 genes), 1.5–1.75 (early; 2698 genes), < 1.5 (late; 3093 genes). Genes that mapped to each bin were then compared to the complete set of ~9000 genes. Results for over and underrepresented functional categories for each replication-timing bin are listed in Table 5 along with the GOstat results for islands. For GO hierarchies where multiple ontologies were overrepresented, the most specific member was listed. Complete results for GOstat analysis of islands and Woodfine replication markers can be found in Additional file 4. It should be noted that there were several functional categories that were overrepresented in the original set of genes that overlapped with Woodfine replication markers (Table 5). These categories were associated primarily with the major histocompatability complex (MHC) that is found on chromosome 6 where the replication timing markers were placed at high density as described previously [18].

As shown in Table 5, there are some overrepresented categories that are found in island DNA but not in any of the bins of replication markers tested in lymphoblastoid cells. One reason for this may simply be the position of replication markers tested by Woodfine et al. [18,19] since these markers were not randomly distributed throughout the genome. For example, only four of the 19 WNT genes overlap with these markers, which would make it difficult to determine whether they were overrepresented in any replication timing bin. It is also possible that the way that we partitioned these markers into bins obscured some GOstat results.

Temporal replication patterns for functionally related genes have been shown previously for the β-globin [55] and immunoglobulin heavy-chain locus gene clusters [5] but not for genes dispersed throughout the genome. The GOstat results in Table 5 give us an indication that there are temporal patterns of replication for some functionally related genes. Apoptotic genes for example, are overrepresented in both islands and the earliest replicating fraction from lymphoblastoid cells. This finding leads us to believe that genes involved in apoptosis represent a subset of genes that have been selected to replicate very early in the S phase. Another example of a functional group of genes that replicate at about the same time are genes involved in nucleosome assembly. These genes replicate predominately in the second quarter of S phase based on replication timing ratios reported by Woodfine et al. [18,19]. This time of replication overlaps with the time when histone genes are being actively transcribed; histone transcription levels increase at the onset of S phase but reach their peak at about mid way through S phase [56].

## Conclusion

In summary, in this work we present a genome-wide method for the identification of sequences that replicate early in the S phase. This method relied on the isolation and cloning of early replicating sequences followed by the end-sequencing and mapping of these clones to the nearly complete human genome sequence. This allowed us to evaluate regions with overlapping clones that are similar in size to an average replicon and therefore should replicate at the same time in S phase. This method has the advantage over microarray studies of replication timing in that there should be less contamination from adjacent sequences when trying to identify genomic features that correlate with time of replication. We found that for the earliest replicating sequences in normal human fibroblasts, there is a positive correlation with open chromatin, gene and exon density, and active promoters. Based on our comparison of replication timing in fibroblast and lymphoblastoid cells, these regions are more likely to replicate at the same time in multiple cell types than sequences that replicate at other times in the S phase. In addition, there are a few subsets of functionally related genes, including those for apoptosis and WNT signaling, that are replicated at this time. All of these data suggest that the chromatin structure/gene composition of this compartment of the S phase is conserved and may be important for the maintenance of cell stability and if defective may confer genetic instability. We are currently in the process of identifying origins of replication and replicon boundaries in some of these very early replicating regions in order to understand further the processes that orchestrate the order of replication at the beginning of the S phase.

## Methods

### Flow cytometry

NHF1 cells were synchronized and incubated in BrdUrd for 24 hrs in the presence of aphidicolin as described above. After collection by trypsinization, cells were washed and resuspended in cold saline, fixed in 67% ethanol, then stained with propidium iodide and fluorescein isothiocyanate (FITC)-conjugated anti-BrdU antibody (Becton Dickinson, Franklin Lakes, NJ) as described [57]. Flow cytometric analysis was done on a FACscan (Becton Dickinson) and the number of S phase cells was quantified using WinMDI program (Joseph Trotter, Scripps laboratory, [58]).

### End sequencing of library clones

Cosmid DNA template was purified in 96-well format using LigoChem ProPreps, with ProCipitate, a synthetically engineered protein-binding polyelectrolyte (modification of method described in Kelley et al. [59]). Cycle sequencing was performed using ABI Big Dye Terminator cycle sequencing kits and reactions were run on ABI 3700 or ABI 3730 sequencers.

### Mapping of paired cosmid clone ends to the human genome sequence

Reads were trimmed to minimize those of low quality (Phred score < 20) and to remove vector sequences prior to their alignment to the genome. Trimmed sequences were aligned to the NCBI build 35 (April 2004) sequence assembly using BLAT [28] with the default parameters except ooc = 11. ooc and -stepSize = 5. Resulting alignments were filtered to retain only the best alignments, requiring at minimum 80% of the trimmed sequence aligning with at least 90% base pair identity. Alignments from the 5' and 3' ends of the same cosmid clone were paired when these alignments were on the same chromosome, in the proper orientation, at a distance of at least 30 kb and no more than 50 kb from each other, consistent with sizing that is expected with cosmid cloning. These paired ends delimit the full extent of the clone in the genome sequence. Unpaired end sequences, where the pairing end sequence was either not available, could not be aligned, or was not a correct distance or orientation, were also retained as "orphan" ends. Only clones uniquely placed by paired ends or an orphan end were considered for further analysis. In addition, clones that mapped to nearly the same location as another clone and originated from an adjacent well in the 96-well plates used for sequencing, were removed as probable contaminants.

### Determination of early replicating islands

Positions of early replicating clones were merged to create "islands" of early replication. Only clones within 100 kb of each other were merged, and the resulting island could be no longer than 175 kb. At least two clones were required to form an island.

### Extraction of genomic features

Additional file 2 lists all genomic features considered in this analysis. Except for gene and promoter counts, chromosome bands, and chromatin status, the percentage of each feature was determined for each specified genomic region. These percentages were normalized to a 100-kb window to allow for comparison. Chromatin status was considered only for those genomic regions that overlapped a clone assayed in Gilbert et al. [22].

### Determination of features significantly different in two datasets

For a feature represented by real-valued numbers for each genomic region coming from one of two distinct sets, the non-parametric Mann-Whitney/Wilcoxson ranksum test was used to calculate *z*-scores, providing a measure of the difference of the content of that feature in the two sets. To determine whether this difference was significant, 10,000 balanced permutations tests [60] were performed. Those features where no more than one of the 10,000 permutations had greater z-scores were considered significantly different in the two sets of sequence regions.

### Cell cultures, synchronization of normal human fibroblasts and isolation of replicating DNA

NHF1-hTERT cells, the immortalized cell line derived from NHF1 cells by ectopic expression of the catalytic subunit of telomerase [61], were used for these studies. NHF1-hTERT cells were grown in Dulbecco's modified essential medium containing 2X the concentration of MEM non-essential amino acids (GIBCO-BRL, Grand Island, NY). Growth media was supplemented with 2 mM L-glutamine (GIBCO-BRL) and 10% fetal bovine serum (HyClone Laboratories, Inc., Logan UT).

Cells uniformly labeled with ^14^C-thymidine were synchronized by a combination of confluence arrest, followed by replating at lower cell density and treatment with aphidicolin for 24 hr. After removal of the inhibitor, the cells progress through the S phase as a synchronous cohort. The synchronized cells were labeled with BrdUrd and ^3^H-thymidine during sequential 1-h periods of the S phase and the replicated, hybrid-density DNA was fractionated by CsCl gradient centrifugation [32,62]. After dialysis and concentration, the total amount of replicated DNA in the samples was determined from the specific activity of ^14^C-labeled DNA.

### Determination of replication timing in Brdu-labeled nascent DNA

DNA replicated in different windows of the S phase was tested for the relative abundance of selected genetic markers (Table 2). PCR primers were designed using Primer3 [63] or Prime (GCG Version 11.0, Accelrys Inc., San Diego, CA). PCR was performed using the OmnE and PCR Express thermocyclers (Thermo Electron Corporation, Waltham, MA) with Thermo-Start DNA polymerase (ABGene inc., Rochester, NY). PCR reaction conditions and, capture and analysis of digital gel images were described previously [31]. Equal amounts of replicating DNA from samples representing seven hrs of the S phase were used as template for PCR reactions. In order to obtain quantitative results, it was necessary to carry out the PCR reactions under non-saturating conditions, and to construct a standard curve with genomic DNA of the same size as the tested DNA [31,32]. DNA used for these PCR standard curves consisted of purified human genomic DNA, which was sheared 10 times through a 21-gauge needle, in the same way as the BrdU-labeled DNA prior to CsCl gradient centrifugation [62]. Table 2 lists data from all markers tested. A numerical value approximating the time of replication was determined for each PCR marker tested using a previously described weighted average method [32]. Briefly, a synchronized population of cells travels as a cohort through the S phase with the broadness of the peak reflecting the degree of synchrony. Therefore, it is necessary to consider more than one hourly replication fraction when calculating replication time. In order to determine the replication timing for each PCR marker, we first normalized by assigning the value of one to the highest sample and scaling all the other measurements to their corresponding fraction. Then, we included in our weighted average analysis the peak replication fraction and any other fraction that had > = to 50% of the peak value. This method while not producing an exact replication time does provide a good estimate, and allows for comparison among markers.

## Abbreviations

SINE, short interspersed repeat element; LINE long interspersed repeat element; LTR, long terminal repeats; NHF1, normal human fibroblasts; BrdUrd, bromodeoxyuridine; MIR, mammalian-wide interspersed repeats; MaLR, mammalian LTR; ERV1, endogenous retrovirus 1; ERVL, endogenous retrovirus L; WNT, wingless-type MMTV integration site family.

## Authors' contributions

SC performed the PCR based replication timing studies, analyzed the results of the GOstat analysis, and drafted the manuscript. TF carried out all of the bioinformatics analyses, alignment of clone sequences, identified early replicating islands and participated in the drafting of the manuscript. ND was responsible for end-sequencing the library clones. DK was the initiator and director of this project. All authors participated in the design of this study and approved the final manuscript.

## Supplementary Material

Additional file 1 Analysis of tandem repeats associated with clone islands. Click here for file

Additional file 2 List of all genomic features analyzed. Click here for file

Additional file 3 Genes associated with clone islands. Click here for file

Additional file 4 Results of GO database analysis using the GOStat program available at . Click here for file

## Figures and Tables

**Table 1:** Distribution of islands.

**Chromosome**	**Percent Enrichment**^1^	**Mean Replication Timing Ratio**^2^
19	198%	1.72
17	183%	1.64
16	175%	1.56
22	160%	1.75
15	123%	1.57
10	120%	1.49
11	113%	1.49
1	112%	1.52
7	110%	1.45
20	101%	1.60
12	97%	1.50
9	96%	1.44
8	89%	1.39
6	82%	1.44
14	82%	1.46
18	79%	1.42
2	79%	1.43
5	73%	1.42
3	71%	1.43
21	66%	1.42
4	55%	1.34
13	48%	1.36
X	28%	1.38
Y	25%	1.32

1- The percentage of total DNA in islands that originated from each chromosome was determined and compared to the percentage of genomic DNA for each chromosome.

2- Mean replication timing ratio for each chromosome as reported in Woodfine et al. [19]. Higher numbers indicate an earlier average time of replication.

**Table 2:** PCR markers analyzed and their replication timing.

**Primer set name**	**Sequence**	**Product range**	**Replication time in hrs**^**1 **^**(fibroblasts)**	**Woodfine replication timing ratio**^**2**^
chr6.1385A	F ctcagctttccctgttaatg R ggaagatgctaaatgactgc	30904535–30904712	1.0	1.73
chr6.1385B	F ggaattaaggcgtgtatctg R aacttcccatagggattagc	31023340–31023694	1.5	1.63
chr6.1386A	F agtaaatccgggtctctagg R ccagatagaggcactgagag	32021221–32021449	1.0	1.74
chr6.1386B	F aaatgtccttcaccatcaag R attatcagagcagcaaaagg	32151938–32152179	0.9	1.74
chr6.1395A	F atgactcatgtagggcagac R aaggaggaacaagaggaaac	40489161–40489555	5.6	1.36
chr6.1395B	F gtccaagctaagtccatgag R gatgacaaacatgaatgcag	40560441–40560763	5.5	1.25
chr6.1395C	F taggtcagttgacccatctc R cgtgttcagtgtgtatttgc	40601430–40601688	5.4	1.58
chr6.1401A	F caggcccatacttcttgtag R gtgagagcttctccttgatg	43125237–43125402	1.6	1.73
chr6.1401B	F tggttcttctctccagtttg R taggtgaggacacaagatcc	43237884–43238178	2.1	1.73
chr6.1417A	F cagtagtgtgtgcacctgtc R tccctgtaacttcaaccatc	89898270–89898634	4.6	1.51
chr6.1417B	F taaccccactctgtgttagg R cctatggagtcagagattgc	89963481–89963615	4.8	1.51
chr22.1096A	F agggtcacatctacagttgg R ccatttgctgtcctttctac	23123485–23123611	1.1	1.85
chr22.1096B	F aatcctcctgagaattaggc R caggagtaatgcccacttag	23239758–23239902	0.5	1.72
chr22.1102A	F tcaggaggagtttcacattc R cacagaagactcagggagag	26718830–26718968	1.0, 5.0	1.58
chr22.1102B	F gctaggacctgaactcacac R tttaaagggctgctctattg	26841004–26841279	5.6	1.55
chr22.1110A	F gccctgagatactgactctg R aaatgcagaatgaagtggag	31334872–31335235	1.0	1.55
chr22.1110B	F gacattgctcttgcctctac R cttctcaagggtgtaaatgg	31441419–31441684	1.0	1.54
chr22.1112A	F caggcatctgaaattaaacc R agattagaggctggttttcc	32486488–32486736	6.0	1.68
chr22.1112B	F actgagattcaaaccagagc R agggaaacttggtaatagcc	32533028–32533232	6.1	1.64
chr22.1118A^3^	F ttcagggtctggttggtagg R agcaggagactggcacagat	38071800–38072021	0.8	1.99
chr22.1118B	F gtggggtgcactgttactat R atcagggacacgtaaacaac	38181333–38181482	0.8	1.95

1- Data derived from the average replication time from two synchronizations.

2- Data derived from lymphoblastoid cells (Woodfine et al. [18, 19]).

3- Primer is located 25 kb upstream of island in an adjacent clone.

**Table 3:** Genomic features significantly different in islands compared with whole genomic DNA.

			**Islands that overlap with replication timing markers**^**1**^		
			
**Feature**	**Genome**	**All Islands**	**Islands with a ≥ 1.65-timing ratio**^**1**^	**Islands with a ≥ 1.75-timing ratio**^**1**^	**Islands with a ≥ 1.85-timing ratio**^**1**^
**100-kb windows**	28358	1749	228	144	78
**Genes**	30078	2815	606	428	252
**Genes/100 kb**	1.06	1.60	2.66	2.97	3.23
**Promoters**^**2**^	10415	948	223	170	111
**Promoters/100 kb**	0.37	0.55	0.98	1.18	1.42
					
**Transcribed**	37.61%	43.73%	56.37%	55.88%	54.79%
**Translated**	1.11%	1.81%	3.08%	3.42%	3.35%
**CpG_Islands**	1.94%	4.05%	5.20%	5.83%	6.58%
**CpG_Content**	1.96%	2.76%	3.39%	3.63%	3.86%
**GC_Content**	40.88%	45.51%	48.71%	49.82%	50.52%
**Conserved**	4.83%	5.78%	7.18%	7.50%	7.86%
					
**All_Repeats**	48.52%	45.68%	44.23%	44.78%	46.21%
**SINE**	13.67%	17.18%	23.28%	25.05%	27.71%
**Alu**	10.75%	13.54%	18.99%	20.66%	23.59%
**MIR**	2.92%	3.64%	4.29%	4.39%	4.11%
**LINE**	21.10%	15.94%	11.55%	10.73%	10.06%
**L1**	17.48%	12.27%	7.83%	6.97%	6.53%
**LTR**	8.71%	7.45%	5.28%	4.98%	4.65%
**MaLR**	3.79%	3.30%	2.37%	2.34%	2.18%
**ERV1**	3.00%	2.58%	1.76%	1.66%	1.51%
**ERVL**	1.61%	1.32%	0.85%	0.75%	0.71%
**DNA transposons**	3.02%	2.71%	2.35%	2.23%	2.02%
**MER2_type**	1.06%	0.79%	0.63%	0.55%	0.54%
**Mariner**	0.10%	0.07%	0.04%	0.04%	0.03%
**Low_complexity**	0.58%	0.54%	0.51%	0.50%	0.49%
**Tandem_Repeats**	1.82%	2.64%	2.1%	2.24%	2.44%

1- Replication timing ratio determined by Woodfine et al. [19].

2- Active promoters in IMR90 fibroblasts as determined by Kim et al. [44].

**Table 4:** Chromatin structure and replication timing. Chromatin ratios were determined by Gilbert et al. (2004).

	**100-kb windows**	**Average Chromatin Ratio**	**Regions of Open Chromatin**
**Genome**	6852	-0.0396	3230 (47.1%)
**All Islands**	461	0.3482	309 (67.0%)
**Islands with a ≥ 1.65-timing ratio**^**1**^	194	0.6374	158 (81.4%)
**Islands with a ≥ 1.75-timing ratio**^**1**^	130	0.7983	112 (86.1%)
**Islands with a ≥ 1.85-timing ratio**^**1**^	73	0.8993	66 (90.4%)

1. Replication timing ratio determined by Woodfine et al. [19].

**Table 5:** GOstat results. Representation of different GO functional categories as determined by the GOstat program [48].

		**Woodfine replication markers**^**1**^		
**Function**	**Islands**	**> 1.75**	**1.75–1.5**	**< 1.5–1.25**
Apoptosis (programmed cell death)	over	over		
Frizzled-2 (WNT) signaling pathway	over			
Intracellular ligand-gated ion channel activity	over			
Transmembrane receptor protein tyrosine kinase activity	over			
Transmembrane receptor protein tyrosine phosphatase activity	over			
G-protein coupled receptor activity	under	under		over
Rhodopsin-like receptor activity	under	under		over
G-protein coupled receptor protein signaling pathway	under	under	under	over
Olfactory receptor activity	under		under	over
Sensory perception of smell	under		under	over
Proteasome complex (sensu Eukaryota)		over		
Cation-transporting ATPase activity		over		
Positive regulation of signal transduction		over		
Lipid biosynthesis		over		
Lipid metabolism		over		
Intracellular protein transport		over		
Receptor signaling protein activity		over		
Regulation of transcription factor activity		over		
Protein binding^2^		over		under
Positive regulation of cellular process		over		under
Intracellular membrane-bound organelle		over		under
Cell surface receptor linked signal transduction		under	under	over
Transmembrane receptor activity		under		over
Neurophysiological process		under		over
Nucleosome assembly		under	over	
DNA binding			over	
DNA metabolism			over	
Antigen processing endogenous antigen via MHC class I^2^			over	
MHC class I protein complex^2^			over	
MHC class I receptor activity^2^			over	
Calcium-mediated signaling			over	
Calcium ion binding			under	
Carbohydrate binding			under	
Muscle contraction			under	
Integral to membrane			under	over
Phosphoric ester hydrolase activity				over
Organismal physiological process				over
Cell communication				over
Glutamate signaling pathway				over
Keratin filament				over
Sensory perception of taste				over
Positive regulation of cellular metabolism				under

1- Replication timing ratios determined by Woodfine et al. [18, 19].

2- Indicates functional categories overrepresented in genes associated with Woodfine et al. ([18, 19]) timing markers.
